# *In vitro* cytotoxic and pro-apoptotic effects of *Lactobacillus* rhamnosus GG cell-free supernatant on HT-29 colorectal cancer cells

**DOI:** 10.3389/fmolb.2026.1868859

**Published:** 2026-09-17

**Authors:** F. Fareedhul Fahmitha, Manal Mowaffaq Mohammed, Prithviraj Karak, Estabraq Ali Hameed, Basim Mohammed Hanon, Israa M. Essa, Hasanain A. J. Gharban, K. Gayathri, P. Saranraj, M. Manigandan, Afsona Parveen, Sayandeep K. Das

**Affiliations:** 1 PG and Research Department of Microbiology, Sacred Heart College (Autonomous), Tirupattur, Tamil Nadu, India; 2 Department of Biology, College of Education for Pure Sciences, University of Al-Hamdaniya, Al-Hamdaniya, Iraq; 3 Department of Physiology, Bankura Christian College, Bankura, India; 4 Scientific Affairs Department, University of Al-Hamdaniya, Nineveh, Iraq; 5 Department of Microbiology, College of Veterinary Medicine, University of Wasit, Wasit, Iraq; 6 Department of Public Health, College of Veterinary Medicine, University of Basrah, Basra, Iraq; 7 Department of Internal and Preventive Veterinary Medicine, College of Veterinary Medicine, University of Wasit, Kut, Iraq; 8 School of Allied and Healthcare Sciences, Centurion University of Technology and Management, Jatni, Odisha, India; 9 Department of Pathology, Shri B. M. Patil Medical College Hospital and Research Centre, BLDE (Deemed to be University), Vijayapura, Karnataka, India

**Keywords:** apoptosis assay, cell cycle, GC-MS analysis, HT-29 cell line, *Lactobacillus* rhamnosus GG, MTT assay

## Abstract

**Background:**

Colorectal cancer is a leading cause of cancer-related mortality worldwide, underscoring the need for effective therapies with lower toxicity. Probiotic-derived metabolites (postbiotics) have emerged as promising anticancer components with immunomodulatory and antiproliferative effects.

**Methods:**

Cell-Free Supernatant (CFS) from *Lactobacillus rhamnosus* GG, produced under microaerophilic conditions and characterized by GC-MS, was tested against HT-29 colorectal adenocarcinoma cells. Cytotoxicity was evaluated by MTT assay, apoptosis following Annexin V-FITC/PI flow cytometry, and cell cycle distribution.

**Results:**

Growth under microaerophilic conditions yielded a sterile CFS that was subsequently profiled by GC-MS. Cytotoxicity was assessed by MTT assay, while apoptosis and cell-cycle distribution were evaluated by flow cytometry. GC-MS showed more than 65 chromatographic peaks, and 28 major compounds were tentatively identified by NIST-library matching; diketopiperazines accounted for approximately 45% of the total identified peak area. The study-strain CFS reduced HT-29 cell viability in a concentration-dependent manner, with an estimated IC50 of 14.2 micrograms/mL expressed as total lyophilized solids. Annexin V-FITC/PI flow cytometry showed 47.6 ± 2.9% early apoptotic, 2.3 ± 0.8% late apoptotic, 2.3 ± 0.6% necrotic, and 47.8 ± 3.2% viable cells after treatment. Treated cells were distributed as 47.3% G1, 38.9% S, 3.22% G2/M, and 2.60% sub-G1; because reliable quantitative control data were unavailable, phase-specific arrest could not be established.

**Conclusion:**

The study-strain CFS showed concentration-dependent in vitro cytotoxic and pro-apoptotic activity against HT-29 cells. The activity may be associated with the partial metabolite profile identified by GC-MS, but specific active compounds and molecular pathways were not established. The cell-cycle findings are descriptive because reliable quantitative control data were unavailable. Further studies using processed-medium controls, non-transformed colonic cells, additional cancer cell lines, orthogonal viability assays, mechanistic markers, and activity-guided fractionation are required.

## Graphical Abstract

**Figure:**
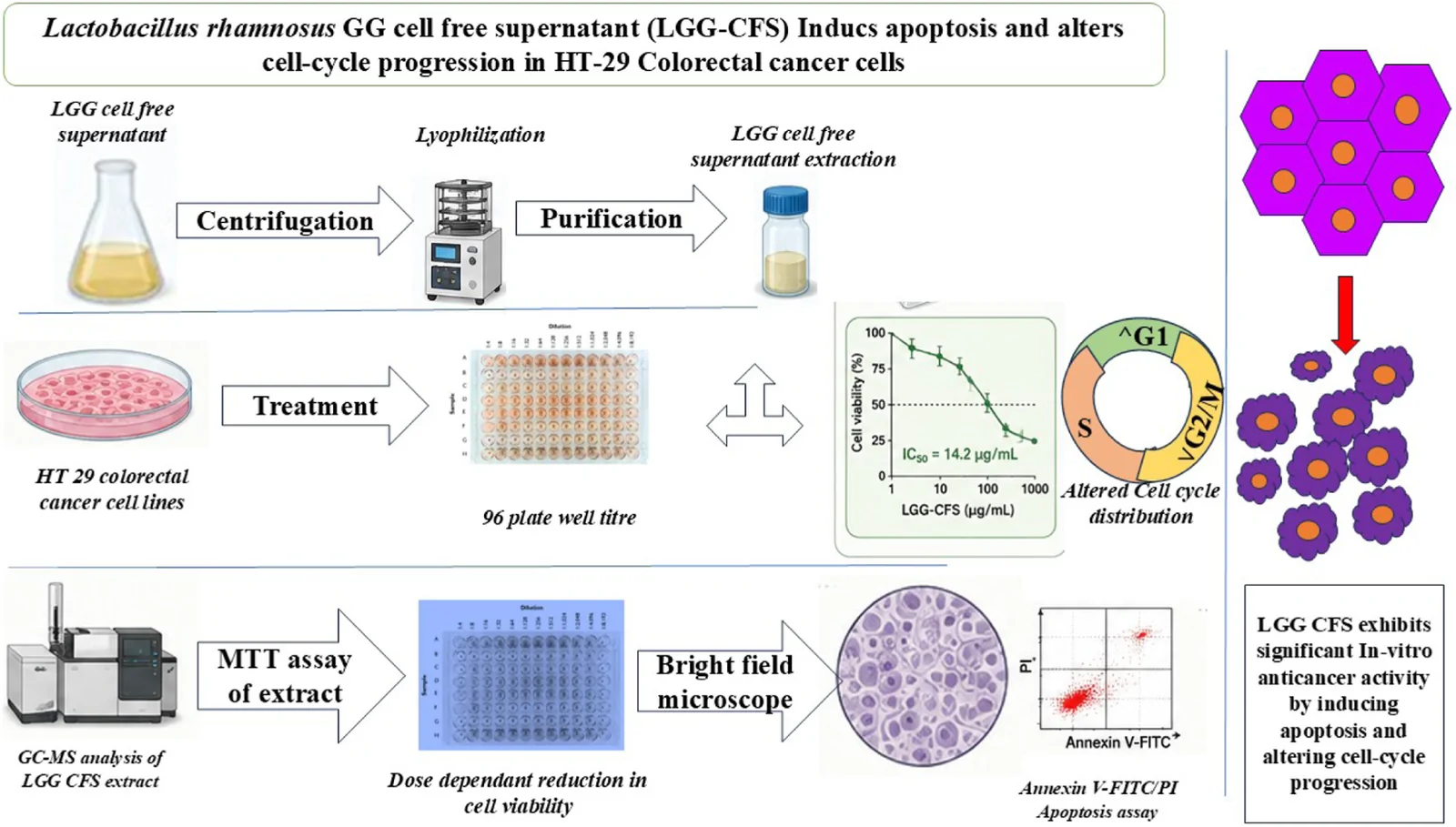

## Highlights

- Colon cancer is a major cause of cancer-related deaths worldwide.
- *Lactobacillus rhamnosus GG* cell-free supernatant (LGG-CFS) exhibits significant *in vitro* cytotoxic and pro-apoptotic effects against HT-29 colorectal cancer cells.
- Probiotic metabolites show promise due to immunomodulatory and anticancer (antiproliferative) properties. However, further validation is required.

## 1 Introduction

The maintenance of host homeostasis depends on the metabolic activity of gut microbiota, which comprises numerous strains of yeast and bacteria. The relationship between human and intestinal bacteria is commensal or mutualistic. The flora of the gastrointestinal tract is anatomical tissue specific; the largest number of species is present in the colon, but the stomach and the small intestine have a relatively low microbiota. In the stomach, anaerobes make up about 99% of the bacteria (Thursby and Juge, 2017). Colon cancer is among the most prevalent malignancies, alongside breast, lung, and prostate cancer. Colorectal cancer often develops through the adenoma-carcinoma sequence. Stage 0 disease is confined to the mucosa; stage I invades the submucosa or muscularis propria; stage II extends through the bowel wall without regional lymph-node involvement; stage III involves regional lymph nodes; and stage IV denotes distant metastasis. Treatment depends on stage, with surgery central for localized disease and systemic therapy used according to recurrence risk and metastatic status (Hossain et al., 2022). Traditional forms of treatment of colon cancer are radiotherapy, chemotherapy, and surgery. For localized colorectal cancer, surgery remains the mainstay of treatment, with neoadjuvant or adjuvant therapy used in selected settings according to stage and clinical context (Xie et al., 2020). Postoperative adjuvant chemotherapy is standard for many patients with colon cancer (stage III), whereas its role in stage II disease remains more selective and contentious (Taieb and Gallois, 2020).

Recent studies have not defined a definite bacterial population, alteration of its relative abundance, or even the number of individual strains that cause increased tumor growth and vulnerability. Studies have reported lower relative abundances of some Clostridiales members, including *Faecalibacterium*, *Blautia*, *Bifidobacterium*, and *Lactobacillus*, in patients with colorectal cancer, whereas *Fusobacterium nucleatum*, *Escherichia coli*, *Bacteroides fragilis*, and *Porphyromonas gingivalis* may be enriched. Some bacteria, such as *Fusobacterium nucleatum, Escherichia coli, Bacteroides fragilis* and *Porphyromonas gingivalis,* are shown to be highly increased in such individuals (Gagnière et al., 2016). However, it is still impossible to ascertain a strongly causal connection, that is, to prove the presence of a causal association of specific microbial populations or their relative abundance with cancer. An example would be when *Clostridium* species are often referred to as onco-microbes (Atarashi et al., 2011). The gut microbiota and interspecies interactions are so complex that they have not yet provided a clear-cut bacterial signature that predicts the development of tumors. The gut microbiota, using its effects that cause inflammation and proliferation, under certain conditions of human genetic factors and variables of context, can be able to either health-promoting or tumor-driven (Drago, 2019).

LGG has been shown to be quite resilient over 3 decades of research, having acid-resistant gastric pH and having a strong adhesive capacity to intestinal lining on the epithelial mucosa, as well as developing protective biofilms, according to Capurso (2019). Moreover, LGG is efficient in protecting non-transformed intestinal epithelial cells against numerous chemicals as well as mechanical abuses and averting the expansion of pathogenic intestinal micro-organisms. Notably, LGG has the ability to improve anti-tumor immune responses and reduce inflammatory states of the gut by initiating a Th1 biased response in the host (Capurso, 2019). Taking advantage of these properties, LGG can be clinically used to alleviate diarrhea, intestinal permeability and mucositis caused by such diseases as cystic fibrosis, non-alcoholic hepatitis, inflammatory bowel disease, *Clostridioides difficile* infection, antibiotic therapy, and even anticancer therapy (Banna et al., 2017). Notably, LGG-derived CFS have been reported to act against the malignant cells relative to non-transformed cells, for example, selective cytotoxicity towards colorectal (HCT-116, HT-29 and Caco-2) and melanoma (A375) cell lines relative to primary human fibroblasts (Salemi et al., 2023), and selective cytotoxicity towards leukemic Jurkat cells relative to normal PBMCs (Banakar et al., 2023).

Although several studies have demonstrated the anticancer potential of *Lactobacillus rhamnosus* GG and its cell-free supernatant against different cancer cell lines, important gaps remain. Most previous investigations have focused primarily on cytotoxicity without simultaneously correlating metabolite profiling with apoptosis and cell-cycle alterations. Furthermore, the chemical composition of LGG-derived post biotic metabolites has not been comprehensively characterized in relation to their potential contribution to anticancer activity. Therefore, the present study aimed to characterize the volatile metabolite profile of LGG-CFS using GC-MS and evaluate its cytotoxic and pro-apoptotic effects on HT-29 colorectal cancer cells.

## 2 Materials and methods

### 2.1 Isolation of *L. rhamnosus* GG

*Lactobacillus rhamnosus* GG (MTCC 1408) was obtained from the Microbial Type Culture Collection and used in all experiments. The strain was aseptically inoculated into MRS broth to revive Lactic Acid Bacteria (LAB). It was then streaked onto the MRS agar plate for purification of isolated colonies. Preliminary phenotypic identification involved Gram staining, biochemical test and microscopic analysis of cell morphology (Figures 1, 2).

**FIGURE 1:**
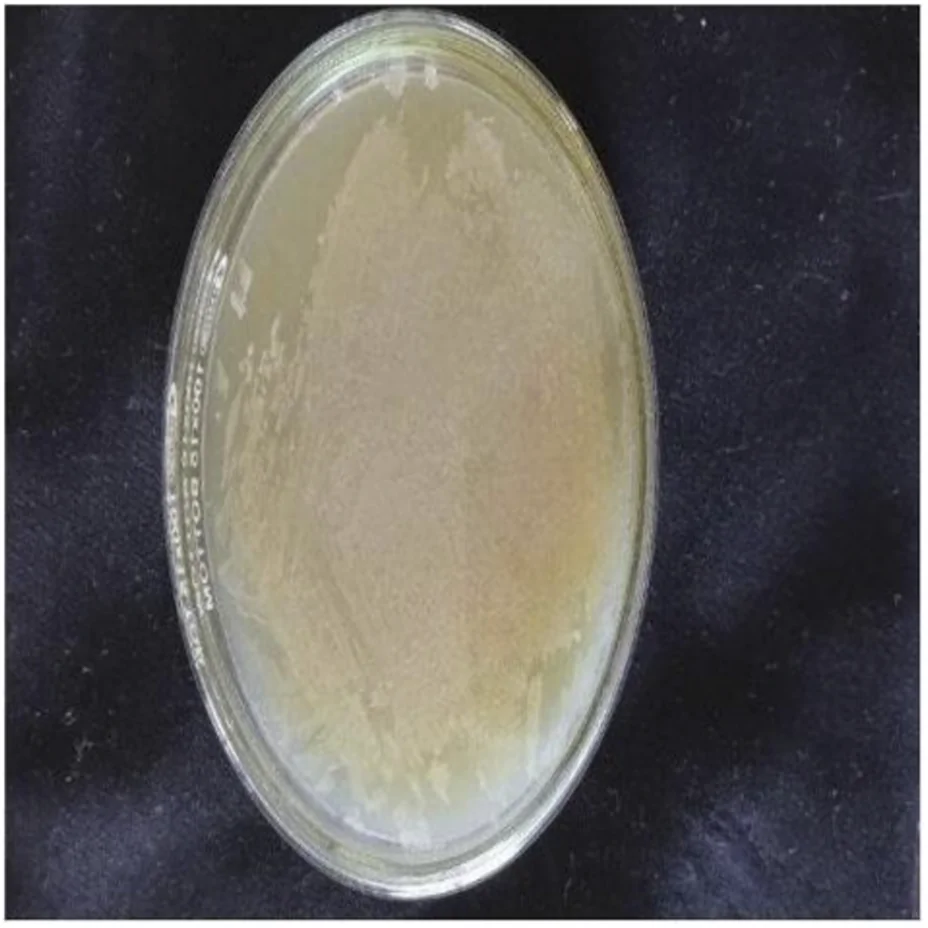
Colony morphology of *L. rhamnosus* GG on MRS agar after 48 h incubation at 37 °C under microaerophilic conditions.

**FIGURE 2:**
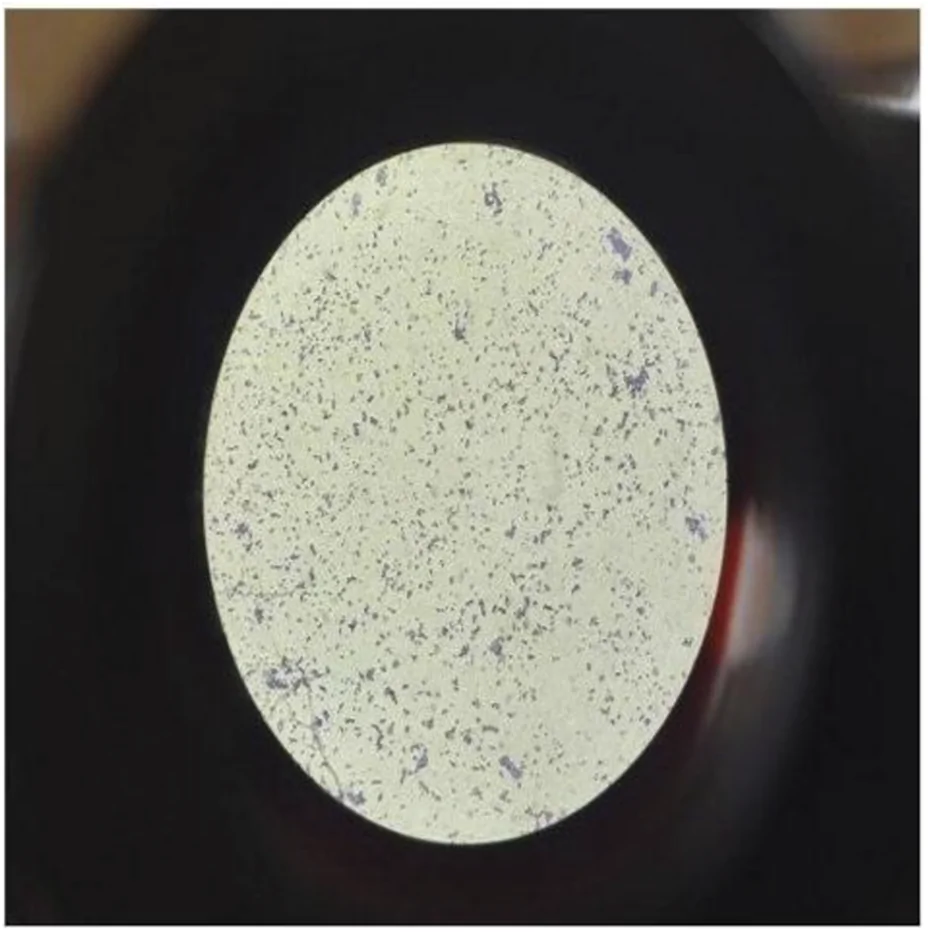
Gram staining morphology of *L. rhamnosus* GG showing Gram-positive chain rods (1000× magnification).

### 2.2 Collection and standardization of CFS from *Lactobacillus rhamnosus* GG

The strain *Lactobacillus rhamnosus* GG (LGG) was initially grown on MRS agar at 37 °C for 48 h under microaerophilic conditions. The seed culture was prepared in microaerophilic culture conditions in MRS broth. Cell pellets were obtained via centrifugation at 8,000 × g for 15 min at 4 °C. The pellets were washed twice with sterile physiological saline solution and then 200 µL of the seed culture was inoculated into 500 mL of MRS broth and incubated for 20 h at 37 °C. Cultures were grown in sterile 1-L Erlenmeyer flasks containing 500 mL MRS broth (50% working volume) to ensure adequate gas exchange. Microaerophilic cultivation was selected because LGG exhibits optimal growth and metabolite production under reduced oxygen conditions, which better mimic the intestinal environment and enhance secretion of bioactive postbiotic metabolites. To control for batch-to-batch growth variability, OD600 was recorded at harvest for each independently prepared CFS batch; only batches within an OD600 range of 1.8–2.1 were used.

Upon completion of the incubation, the culture was subjected to centrifugation at 7,000 × g for 30 min at 4 °C. Approximately 450 mL of recovered supernatant was sequentially passed through sterile disposable 0.22 μm PES syringe filters (25-mm diameter), with each filter processing approximately 40–50 mL before replacement to prevent clogging. To confirm sterility, 100 μL of the filtrate was plated onto MRS agar and incubated at 37 °C for 48 h under microaerophilic conditions; no colony growth was observed in any experimental batch. To prepare a standardized formulation, the sterilized CFS was freeze-dried at −50 °C and 0.1 mbar for 48 h. The obtained lyophilized powder was denoted LGG-CFS-lyo and stored at −80 °C prior to use. Three independently prepared CFS batches were used across three biological replicates.

Concentration normalization: The freeze-dried LGG-CFS preparation consisted of total lyophilized solids obtained from the neutralized, sterile cell-free supernatant. Consequently, the reported concentrations (µg/mL) represent the mass of total lyophilized material, including bacterial metabolites together with residual medium-derived components remaining after filtration and lyophilization. This approach was adopted to ensure reproducible batch-to-batch dosing of the complete post biotic preparation used for all biological assays. Protein concentration and individual metabolite concentrations were not determined in the present study.

In all *in vitro* studies, a working solution of the studied probiotic strain was prepared by dissolving its lyophilizate in sterile RPMI-1640 medium supplemented with 10% (v/v) FBS and 2 mM L-glutamine. The concentration of the preparation was calculated based on the mass of the lyophilizate dissolved to get a 10 mg/mL (10,000 μg/mL) stock suspension. Thus, all reported concentrations (including the IC50 of 14.2 μg/mL) represent total lyophilized solids (including residual medium components) per mL of culture medium. Serial dilutions of the stock with complete RPMI-1640 medium resulted in the following treatment concentrations: 1,000; 500; 250; 125; 62.5; 31.25; 15.62; 7.81; 3.9; and 1.95 μg/mL.

An MRS medium-derived control was used as a parallel control. To prepare this, the medium was handled identically (centrifuging, filtration, freeze-drying, and solubilization in RPMI-1640) to the studied probiotic strain but without inoculation. The control was applied at the same range of concentrations (Ghasemi et al., 2021).

### 2.3 GC-MS characterization

CFS of *Lactobacillus rhamnosus* GG was prepared as per the earlier protocol. First of all, the starter culture was centrifuged at 8,000 × g for 15 min to obtain bacteria-free supernatant and the resulting supernatant was sterilized using filtration through a 0.22 µm membrane filter. In this research, the CFS was prepared from cultures after 20 h of cultivation (late-exponential growth phase) and extracted using liquid-liquid extraction method. Specifically, 50 mL of sterile CFS was adjusted to pH 2.0 using 6 M HCl in order to increase the efficiency of organic acids and cyclic dipeptide recovery. Two extractions with chloroform-methanol (2:1, v/v) solution were done. Afterwards, the combined organic phases were evaporated to dryness under reduced pressure (40 °C) using a rotary evaporator. The dried sample was derivatized with BSTFA (50 µL) with 1% TMCS at 70 °C for 30 min, diluted with 100 µL of hexane and further analyzed by GC-MS using an Agilent 7890B GC equipped with an Agilent 5977B mass selective detector. In this method, separation was carried out using HP-5MS capillary column (30 m × 0.25 mm i. d., 0.25 µm film thickness). Helium served as the carrier gas at a flow rate of 1.0 mL/min. The injection temperature and volume were 250 °C and 1 μL, respectively, whereas the type of the injection was splitless. For oven temperature programming, initial temperature was set to 50 °C for 2 min, ramped up at 10 °C/min to 280 °C and finally kept at the last value during 10 min (total run time 35 min). Mass spectra were registered by electron impact ionization (70 eV) over a mass range from m/z 50 to 600. Ion source, quadrupole, and transfer line temperatures were 230 °C, 150 °C, and 280 °C, correspondingly, whereas the solvent delay was 4 min. Compound identification was performed via mass spectral comparison with the NIST MS Library (version 2.3) using a minimum 80% similarity index. Authentic standards were not used; therefore, identifications are considered tentative. Peak area percentages were calculated by exclusion of solvent and column bleed effects. All analyses were performed in triplicate with relative standard deviations not exceeding 5% for major peaks. A solvent blank was run every 10 samples to monitor contamination and column bleed. It should be emphasized that the GC-MS analysis characterized only volatile and derivatizable low-molecular-weight metabolites. Non-volatile bioactive components including bacteriocins, proteins, peptides, and exopolysaccharides were not detected by this analytical approach.

### 2.4 Cell line collection and preparation

The human colorectal adenocarcinoma cell line HT-29 was procured from the National Centre for Cell Science, Pune, India. The study was conducted following ethical guidelines and was approved by the Institutional Ethics Committee of Sacred Heart College (Autonomous), Tirupattur, Tamilnadu, India (Ref: IEC/SHC/2026/04). Cells were cultured and maintained in RPMI-1640 enriched with 10% heat-inactivated FBS, 2 mmol/l L-glutamine (L-Glut), 100 IU penicillin and 100 μg/mL streptomycin in 5% CO_2_ environment. Cells were inoculated at suitable densities in multi-well plates and permitted to adhere overnight prior to treatment. Upon reaching approximately 70%–80% confluency, culture medium was substituted with new medium containing varying concentrations of the produced LGG CFS. Control experiments were performed with cells in fresh complete medium in pH-adjusted, unconditioned, RPMI-1640 with 10 per cent FBS, thus controlling possible confounding factors that might arise through the acidification of medium by bacterial metabolites (Salemi et al., 2023).

Prior to cell treatment, the pH of LGG-CFS was adjusted to 7.2–7.4 using sterile 1N NaOH and verified with a calibrated pH meter. This neutralization step was performed to minimize confounding cytotoxicity from organic acids produced during bacterial fermentation. However, we acknowledge that a separate acidified medium control was not included; this represents a limitation addressed in the revised discussion. A single 24-h exposure period was selected based on previously published studies investigating LGG-derived post biotics against colorectal cancer cells, which consistently demonstrated measurable cytotoxic and apoptotic responses within this interval. Since the present study was designed as an initial proof-of-concept investigation, additional time-course experiments (12, 48, and 72 h) were beyond its scope and are planned for future work.

### 2.5 Cell viability analysis (MTT assay)

The cytotoxic effect of LGG-CFS-lyo on HT-29 cells was determined by the MTT test (3-(4,5-dimethylthiazol-2-yl)-2,5-diphenyltetrazolium bromide). HT-29 cells were dissociated from the surface of culture flasks in 0.25% trypsin-EDTA solution. The enzymatic activity of the solution was neutralized by addition of complete RPMI-1640 medium and the cell suspension was separated through centrifugation (300 × g, 5 min) followed by re-suspension in fresh culture medium to obtain a homogeneous suspension of the cells. Cell concentration was adjusted to 1 × 10^5^ cells/mL. One hundred and fifty μL of the cell suspension containing approximately 1.5 × 10^4^ cells were plated to each well of the 96-well plates and allowed to attach in RPMI-1640 supplemented with serum overnight at 37 °C in humidified environment with 5% CO_2_.

After overnight incubation, the old medium was carefully discarded and replaced with 100 µL of serum-free RPMI-1640 medium with various concentrations of LGG-CFS-lyo (1.95, 3.9, 7.81, 15.62, 31.25, 62.5, 125, 250, 500, and 1000 μg/mL, see description in Section 2.2). The concentrations were examined in technical triplicate while the blank samples consisted of 100 µL of sterile RPMI-1640 medium per well without any added compound. Background control wells containing CFS at each concentration in RPMI-1640 without cells were included to correct for any color or turbidity contribution from the CFS itself; these values were subtracted from treatment absorbance readings. After 24 h treatment, the medium was removed and 100 µL of 0.5 mg/mL MTT solution was added to each well. Plates were wrapped in aluminium foil and incubated for 4 h at 37 °C in 5% CO_2_. Metabolically active cells reduce yellow dye MTT to form insoluble formazan crystals (Martínez-Maqueda et al., 2015).

The dye was removed and the formazan crystals were dissolved in DMSO with gentle shaking on an orbital shaker for 10–15 min at room temperature. Absorbance was measured at 570 nm with a reference wavelength of 630 nm with the help of a microplate reader. Blank samples consisted of MTT dye without any cells. Viability of the cells was calculated according to the formula:

$$\begin{array}{ll} \text{Cell Viability }\left(\%\right) & =\left(\text{abackground-corrected absorbance of treated cells}/\right.\\ & \;\left.\text{ background-corrected absorbance of control cells}\right)\times 100 \end{array}$$

The half-maximal inhibitory concentration (IC_50_) was calculated by nonlinear regression analysis using GraphPad Prism software (version 8.0), plotting log_10_(concentration) *versus* percentage viability with the log(inhibitor) vs. response (variable slope) model.

### 2.6 Apoptosis analysis by annexin V-FITC/PI staining

The effect of apoptosis induction was investigated using the Annexin V FITC Apoptosis Detection Kit (BD Pharmingen™, Catalog No. 556547) according to the manufacturer’s instructions. After 24-h treatment with LGG CFS (at the calculated IC50 concentration of 14.2 μg/mL) or vehicle control, HT-29 cells adhered to plates and in suspension were collected, washed two times with ice-cold PBS, and diluted in 1× Annexin V binding buffer to achieve cell concentration of 1 × 10^6^ cells/mL. For analysis, 100 µL (1 × 10^5^ cells) were incubated with 5 µL of Annexin V FITC and 5 µL of propidium iodide (PI, 50 μg/mL) for 15 min at RT in the dark. To each tube 400 µL of Annexin V binding buffer were added after staining. Cells were analyzed by flow cytometry within 1 h using the flow cytometer (Beckman Coulter CytoFLEX)with 488 nm argon laser excitation. FITC fluorescence was measured by FL1 channel with 530 nm band-pass filter. PI fluorescence was quantified using FL2 channel with a >600 nm long-pass filter. A minimum of 10,000 events/sample were measured. Compensation was performed using single-stained samples. Data were analyzed using the Flow Jo software v10. The quadrants were drawn based on unstained and stained control cells. The data were presented as the percentage of cells in each of four quadrants: live (lower left, Annexin V^−^/PI^−^), early apoptosis (lower right, Annexin V^+^/PI^−^), late apoptosis (upper right, Annexin V^+^/PI^+^), and necrosis (upper left, Annexin V^−^/PI^+^) (Pozarowski and Darzynkiewicz, 2004).

### 2.7 Cell cycle analysis

Cell cycle distribution was assessed by propidium iodide (PI) staining. After 24 h treatment with LGG CFS (14.2 μg/mL, IC50) or vehicle control, HT-29 cells were collected, washed with PBS, and fixed in 70% ice-cold ethanol overnight at 4 °C. Fixed cells were washed twice with PBS, resuspended in PBS containing 50 μg/mL PI and 100 μg/mL RNase A, and incubated for 30 min at 37 °C in the dark. DNA content was analyzed by flow cytometry (488 nm excitation, 585 nm emission). Cell cycle distribution (sub-G1, G0/G1, S, G2/M) was determined using ModFit LT software. The control sample was assessed using the same analytical workflow; however, quantitative cell-cycle modeling was not considered reliable because of poor model resolution (G1 coefficient of variation >12%)

### 2.8 Statistical analysis

All experiments were performed with three independent biological replicates (n = 3), each with technical triplicates. Data are presented as mean ± standard deviation (SD). Comparisons between two groups were performed using unpaired two-tailed Student’s t-test. Multiple comparisons were analyzed by one-way ANOVA followed by Tukey’s *post hoc* test. IC50 was calculated by nonlinear regression using the log(inhibitor) vs. response (variable slope) model in GraphPad Prism 8.0. Statistical significance was set at p < 0.05.

## 3 Results

### 3.1 Isolation of pure colonies

Pure colonies of *L. rhamnosus* GG showed white color colonies on Man Rogosa De Sharpe agar when incubated and Gram staining and biochemical test were carried out (Table 1).

**TABLE 1:** Preliminary phenotypic identification of *L. rhamnosus* GG.

Test	Results obtained	Expected for *L. rhamnosus*
Gram staining	Gram-positive chain rods	Consistent
Catalase	Negative	Consistent
Oxidase	Negative	Consistent
Glucose fermentation	Positive	Consistent

### 3.2 GC-MS characterization

The GC-MS analysis of CFS from *L. rhamnosus* GG showed a complex mixture of secreted metabolites with over 65distinct peaks observed over the retention time range of 4.6–38.7 min (Figure 3). The number of major compounds determined depending on the matching with NIST library spectra was 28 (similarity index ≥80%), which represents a great diversity of secondary metabolites (Table 2). The peak at 4.609 min showed the highest relative abundance (33.928% of total ion current) but could not be confidently identified (NIST similarity index <80%) and was presumed to represent a column bleed artifact or siloxane contaminant; this peak was excluded from further analysis. The most common chemical type was diketopiperazine derivative (cyclic dipeptides), with a total of about 45% of the total peak area. The most abundant identified compounds are listed (Table 2). GC-MS analysis revealing diketopiperazines as the predominant compounds (≈45% of total identified peak area) in LGG CFS represents a significant finding; however, these identifications are tentative without authentic standards, and causal attribution of bioactivity to specific compounds requires activity-guided fractionation. Peaks 48 and 50 were identified as isomers of Pyrrolo[1,2-a]pyrazine-1,4-dione, hexahydro-3-(2-methylpropyl)-. In NIST library the varying retention times with same compound indicates their isomeric forms. This has been clarified in notes of Table 2.

**FIGURE 3:**
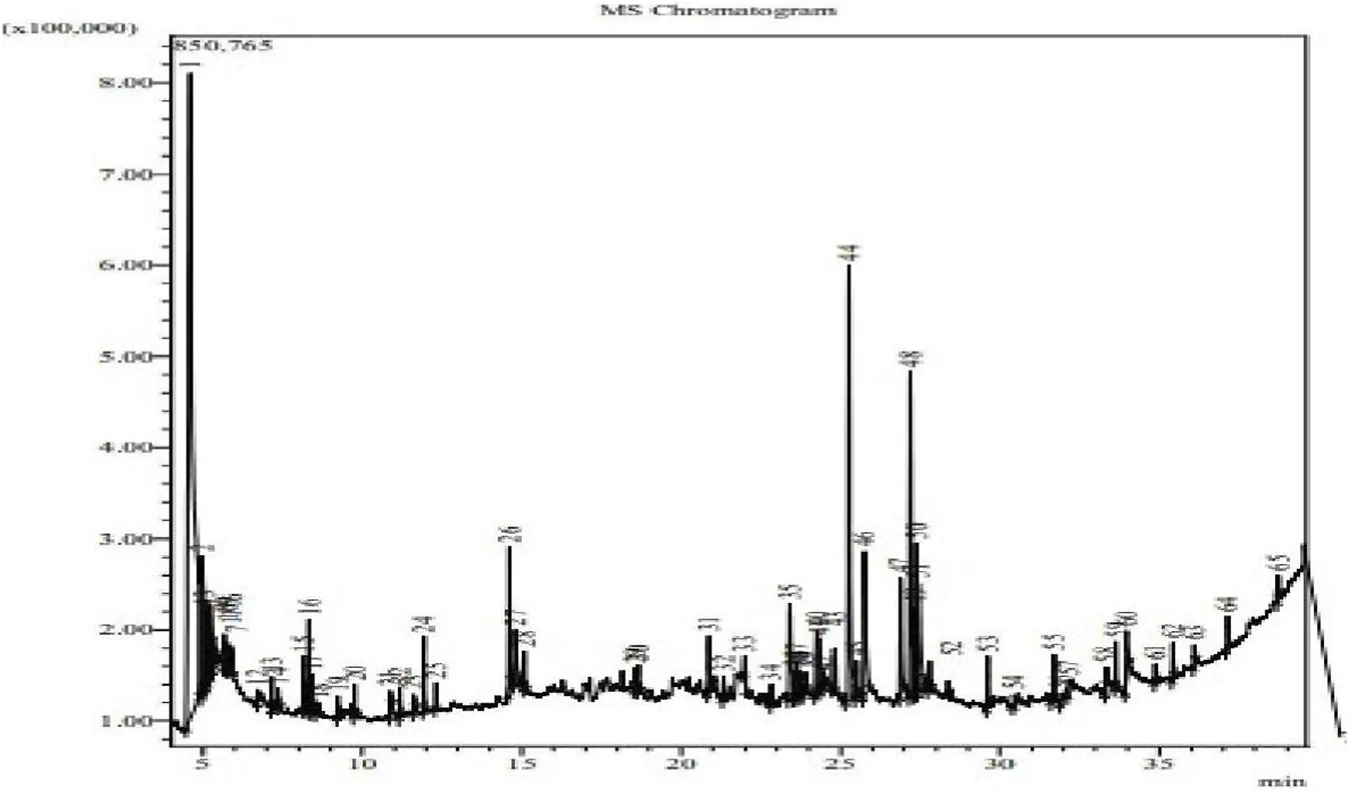
GC-MS chromatogram of *L. rhamnosus* GG CFS. Analysis revealed a complex mixture of secondary metabolites with over 65 peaks distributed across a retention time range of 4.6–38.7 min. The peak at 4.609 min (33.928% of total ion current) could not be confidently identified (NIST similarity index <80%) and is presumed to be a column bleed artifact or siloxane contaminant; it was excluded from quantification. Identified compounds (similarity ≥80%) are listed in Table 2, with diketopiperazines representing ≈45% of the total identified peak area.

**TABLE 2:** Major compounds identified in CFS of *L. rhamnosus* GG (tentative identification by NIST library matching, similarity ≥80%).

Peak #	Compound name	Area %	Retention time	Chemical class
48	Pyrrolo[1,2-a]pyrazine-1,4-dione, hexahydro-3-(2-methylpropyl)-	26.106%	27.205	Diketopiperazine
44	Cyclo(prolylvalyl)	7.608%	25.252	Cyclic dipeptide
51	Hexahydro-3-(1-methylpropyl)pyrrolo[1,2-a]pyrazine-1,4-dione	2.899%	27.444	Diketopiperazine
35	3-Methyl-2,3,6,7,8,8a-hexahydropyrrolo[1,2-a]pyrazine-1,4-dione	2.377%	23.405	Diketopiperazine

Peaks number 48 and 51 were identified as isomers of Pyrrolo[1,2-a]pyrazine-1, 4-dione, hexahydro-3-(2-methylpropyl)- because they exhibited different retention times while sharing the same library identification.

### 3.3 MTT assay

The MTT assay demonstrated significant cytotoxic effect by quantifying cellular metabolic activity as an indicator of viability. A concentration-dependent decrease in cell viability was observed after treatment with the CFS. The cytotoxic activity of LGG-CFS was expressed relative to the concentration of total lyophilized CFS solids. Using this normalization, nonlinear regression analysis yielded an IC_50_ of 14.2 μg/mL. Because this concentration represents the total freeze-dried preparation rather than purified bioactive metabolites or protein content, it should be interpreted as an operational dose for the complete post biotic formulation rather than the concentration of individual active compounds. When the lowest concentration (1.95 μg/mL) was tested, viability remained high at 95.37% with little effect. Viability decreased however, with increases in concentration, to about 60% at 7.81 μg/mL, 50% at 15.62 μg/mL, and below 30% at 125 μg/mL. The optimal effect was observed at the highest concentration (1000 μg/mL) that lowered viability to 14.78%. The dose-response curve observed in Figure 4 is a confirmation that the CFS contains bioactive components, which are effective in disrupting the metabolism of colon cancer cells (Figure 4). Data represent three independent biological replicates (n = 3), each with technical triplicates. Images from all treatment groups were acquired using identical microscope settings, including magnification, illumination, exposure time, and camera gain. Representative bright-field images of HT-29 cells after 24 h treatment are shown in Figure 5. Representative fields were selected without image-specific enhancement. Only uniform brightness and contrast adjustments were applied equally across all images during figure preparation.

**FIGURE 4:**
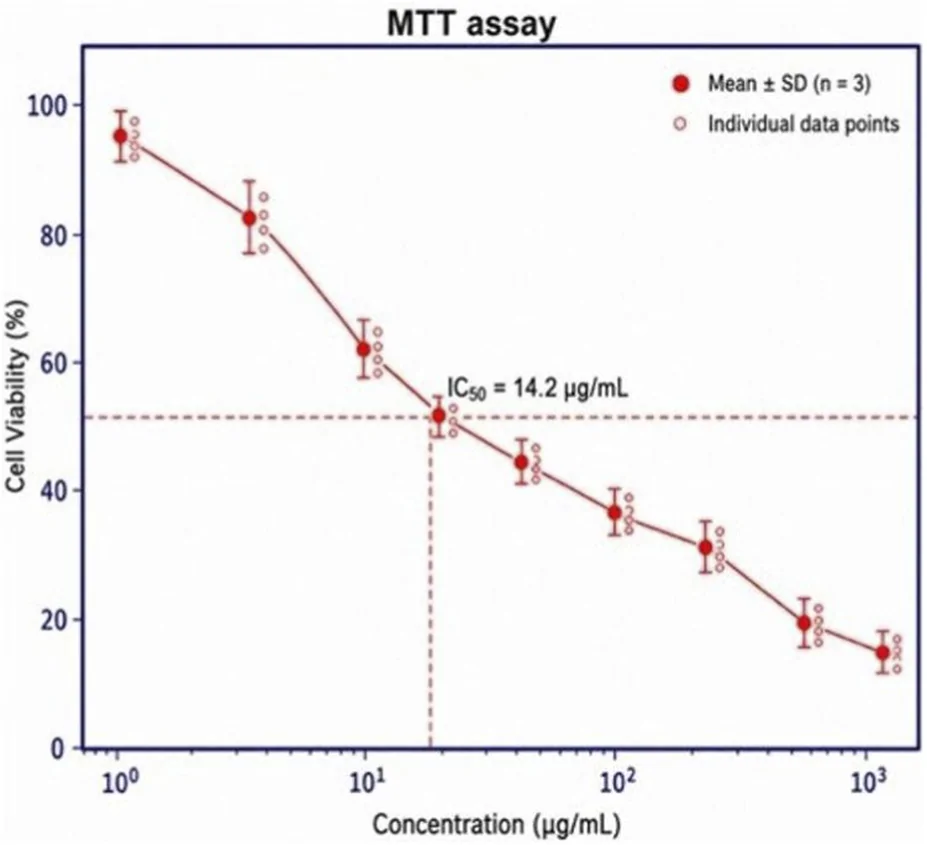
Dose-dependent cytotoxicity of LGG-CFS against HT-29 cells. Cell viability was measured by MTT assay after treatment with increasing concentrations (1.95–1000 μg/mL) for 24 h. Data are mean ± SD from three independent biological replicates (n = 3), each with technical triplicates. The estimated IC_50_ was 14.2 μg/mL of total lyophilized LGG-CFS solids, calculated by nonlinear regression.

**FIGURE 5:**
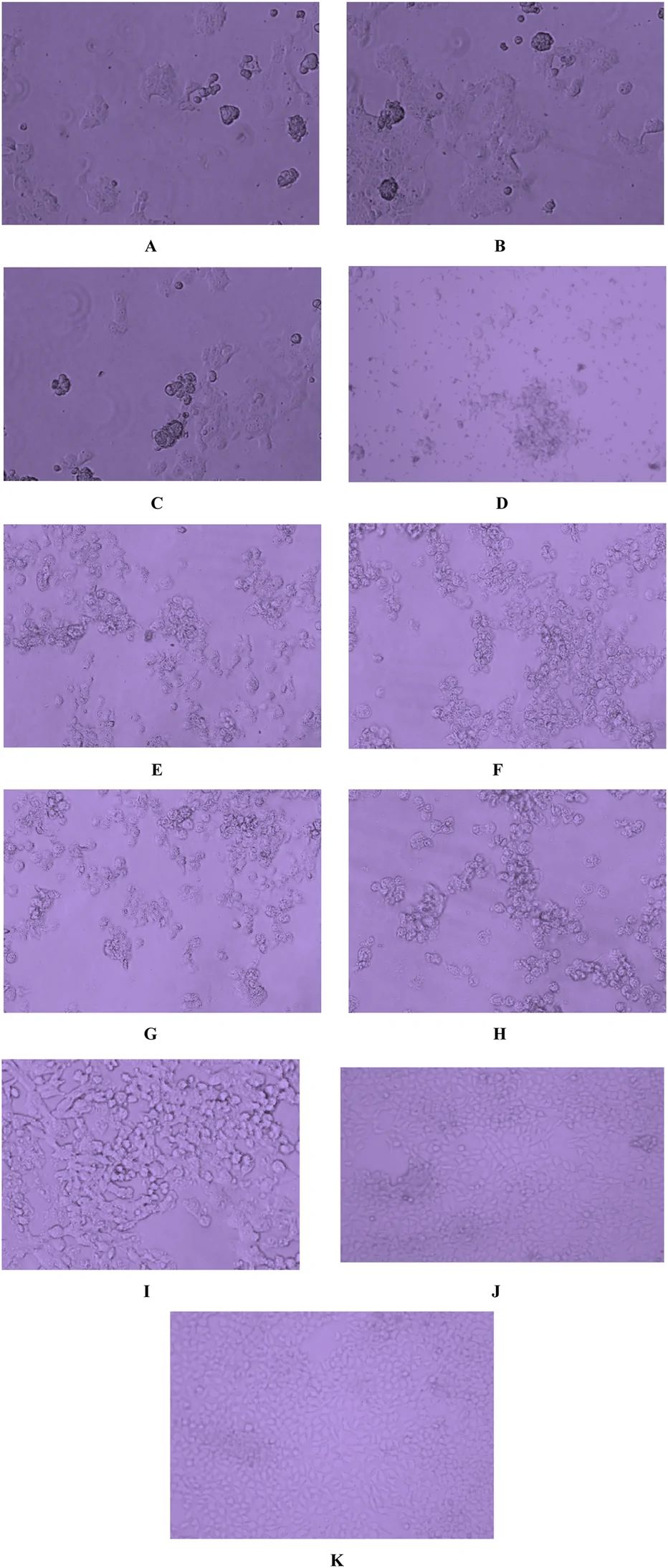
Representative bright-field microscopy of HT-29 cells after 24 h treatment with LGG-CFS. (5K) Untreated control; (5A-5J) treated with increasing CFS concentrations (1000, 500, 250, 125, 62.5, 31.25, 15.62, 7.81, 3.9, and 1.95 μg/mL). Reduced cell density, cell shrinkage, and rounding (morphological features of apoptosis) were observed with increasing CFS concentration. **(A)** 1000 μg/mL **(B)** 500 μg/mL **(C)** 250 μg/mL **(D)** 125 μg/mL **(E)** 62.5 μg/mL **(F)** 31.25 μg/mL **(G)** 15.62 μg/mL **(H)** 7.81 μg/mL **(I)** 3.9 μg/mL **(J)** 1.95 μg/mL **(K)** Control.

### 3.4 Flow cytometric assessment of CFS-induced apoptosis

Apoptosis induction by *Lactobacillus rhamnosus* GG CFS was assessed by Annexin V-FITC/PI dual-staining flow cytometry after 24 h of treatment (Figure 6). The vehicle-treated control (Figure 6A) demonstrated 91.23% viable (Q4; Annexin V^−^/PI^−^), 5.64% early apoptotic (Q3; Annexin V^+^/PI^−^), 2.31% late apoptotic (Q2; Annexin V^+^/PI^+^), and 0.82% necrotic (Q1; Annexin V^−^/PI^+^). This confirmed a healthy baseline population appropriate for comparison with treated samples. Following treatment with LGG-CFS at the calculated IC50 concentration of 14.2 μg/mL (Figure 6B), 47.6% ± 2.9% of cells were distributed in the early apoptotic quadrant (Q3), 2.3% ± 0.8% were late apoptotic (Q2), 2.3% ± 0.6% were necrotic (Q1), and 47.8% ± 3.2% remained viable (Q4).

**FIGURE 6:**
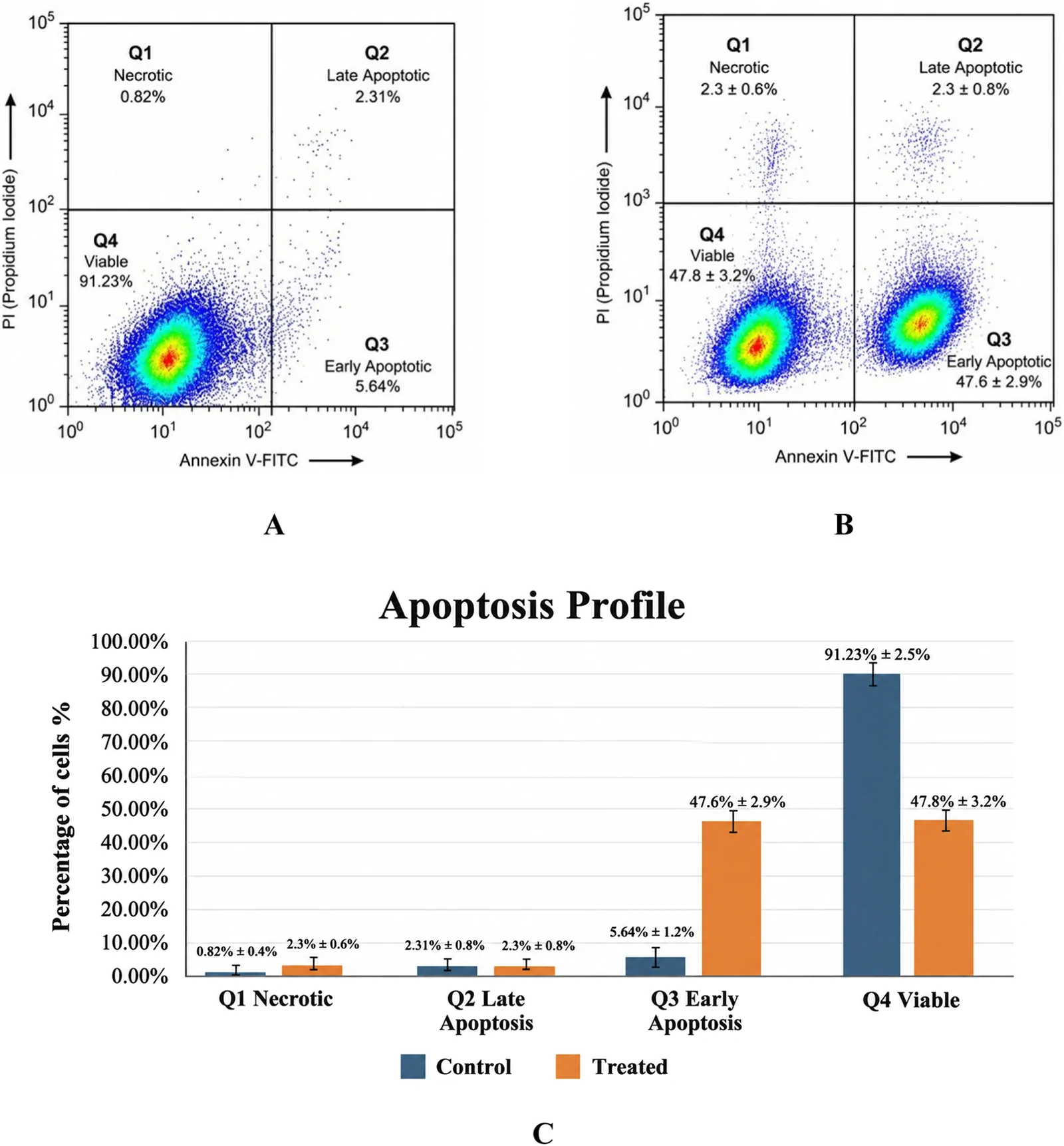
LGG-CFS induces apoptosis in HT-29 cells. Representative Annexin V-FITC/PI dual-staining dot plots after 24 h treatment. **(A)** Vehicle-treated control cells showing 91.23% viable (Q4; Annexin V^−^/PI^−^), 5.64% early apoptotic (Q3; Annexin V^+^/PI^−^), 2.31% late apoptotic (Q2; Annexin V^+^/PI^+^), and 0.82% necrotic (Q1; Annexin V^−^/PI^+^) cells. **(B)** LGG-CFS-treated HT-29 cells (14.2 μg/mL, IC_50_, 24 h) showing 47.8% ± 3.2% viable (Q4), 47.6% ± 2.9% early apoptotic (Q3), 2.3% ± 0.8% late apoptotic (Q2), and 2.3% ± 0.6% necrotic (Q1) cells. **(C)** Bar graph comparing the distribution of necrotic, late apoptotic, early apoptotic, and viable cell populations between control and LGG-CFS-treated groups. Data represent mean ± SD (n = 3).

### 3.5 Cell cycle analysis

Cell-cycle distribution was assessed following 24 h treatment with LGG-CFS (14.2 μg/mL, IC_50_) by propidium iodide (PI) staining and Mod Fit LT analysis. The untreated control histogram showed poor model resolution, with a G1 coefficient of variation (CV) > 12%, precluding reliable quantitative cell-cycle modeling. Therefore, the control profile is presented for qualitative reference only, and no statistical comparison between control and treated groups was performed. In LGG-CFS-treated cells, the observed distribution was 47.30% in the G1 phase, 38.90% in the S phase, 3.22% in the G2/M phase, and 2.60% in the sub-G1 fraction (Figure 7). The predominance of cells in the G1 and S phases suggests an alteration in cell-cycle progression following LGG-CFS treatment. However, because reliable quantitative control data were unavailable, these findings are interpreted descriptively and do not provide definitive evidence of G1/S checkpoint disruption. The relatively low sub-G1 fraction despite the high proportion of Annexin V-positive early apoptotic cells may reflect the 24-h treatment time point, as early apoptotic cells may retain relatively intact DNA and therefore may not be detected in the sub-G1 fraction.

**FIGURE 7:**
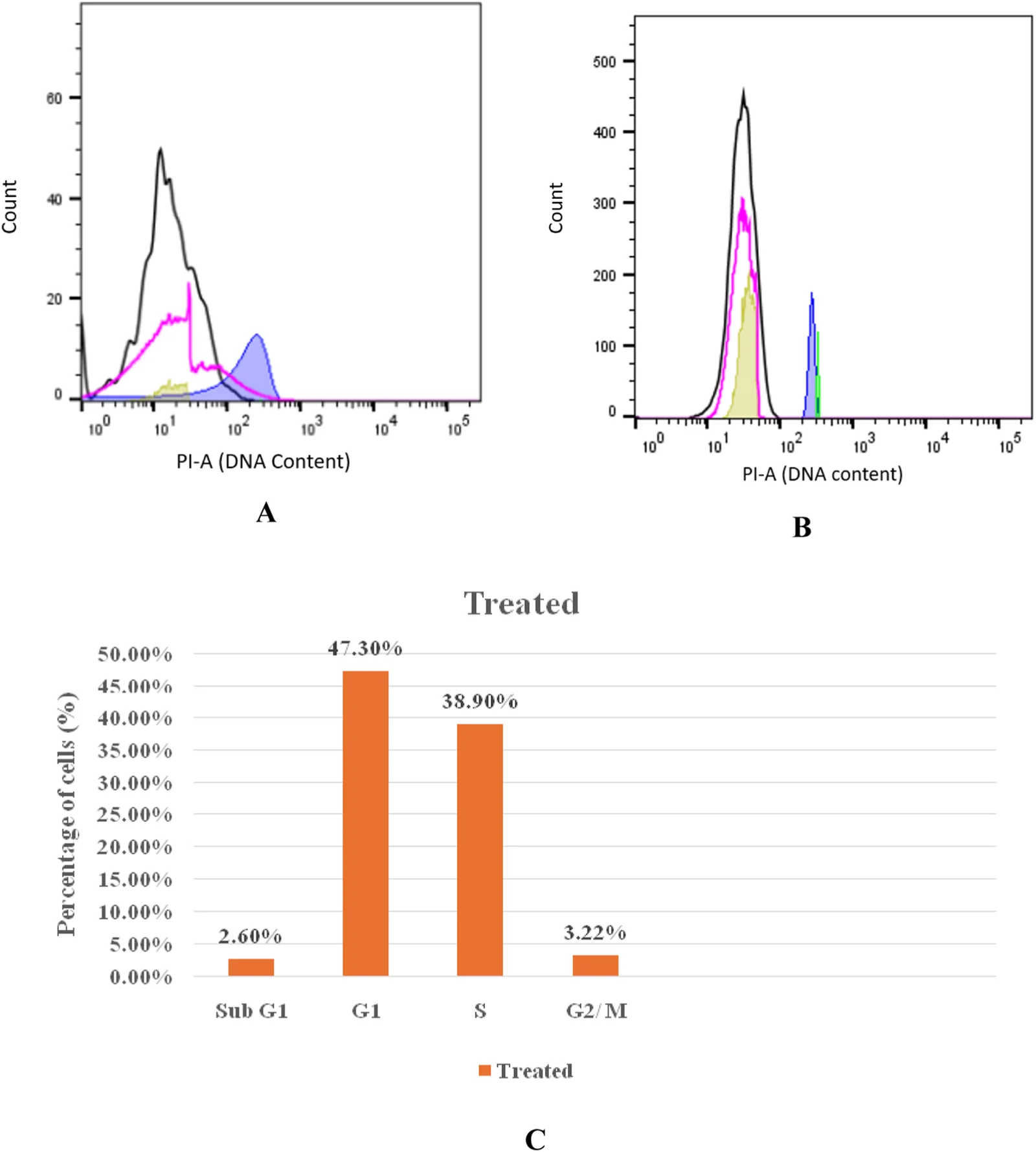
Cell-cycle distribution of LGG-CFS-treated HT-29 cells. **(A)** Representative DNA-content histogram of untreated HT-29 cells stained with propidium iodide (PI), presented for qualitative reference only. Quantitative phase distribution was not reported because the control histogram showed poor model resolution (G1 coefficient of variation >12%). **(B)** Representative DNA-content histogram of HT-29 cells treated with LGG-CFS (14.2 μg/mL; IC_50_) for 24 h, showing 47.30% of cells in G1, 38.90% in S, 3.22% in G2/M, and 2.60% in the sub-G1 fraction. **(C)** Quantitative distribution of treated cells among cell-cycle phases. The observed distribution suggests an alteration in cell-cycle progression following LGG-CFS treatment; however, definitive conclusions regarding G1/S checkpoint disruption cannot be drawn because reliable quantitative control data were unavailable. Data are presented as mean ± SD (n = 3).

## 4 Discussion

The phenotypic characterization of *L. rhamnosus* GG showing Gram-positive chain rods, catalase-negative, oxidase-negative, and positive fermentation of glucose and mannitol aligns with established probiotic profiles (Salemi et al., 2023). Salemi et al. utilized the same ATCC 53103 strain in their investigation of LGG cell-free supernatant as an anti-cancer adjuvant, confirming that this strain demonstrates robust survival at acidic gastric pH, firm adherence to intestinal mucosa, and production of epithelial-protective biofilms. The phenotypic characteristics observed in the current study are consistent with previous reports on LGG (Capurso, 2019). The non-motile, non-spore-forming nature of LGG observed in this study corresponds with the characterization by Morita et al. (2009), who completely sequenced the LGG genome and confirmed its taxonomic position within the *Lactobacillus rhamnosus* species. The negative results for catalase and oxidase are typical for homofermentative lactobacilli that rely on fermentative metabolism rather than respiratory chains (Segers and Lebeer, 2014).

A limitation of this study is the absence of a pH-matched control (e.g., acidified MRS medium neutralized to the same pH), which would help definitively attribute the observed cytotoxicity to specific metabolites rather than residual acidity. Future studies should incorporate such controls.

Liu et al. identified 16 structurally characterized cyclic dipeptides in *Lactobacillus plantarum* (Liu et al., 2025a). Besides cyclic dipeptides, some organic acids and esters were found such as hexadecanoic acid, octadecanoic acid and different methyl esters. Aromatic and heterocyclic compounds were also observed in the GC-MS profile such as indole compounds, pyrazines, naphthalene derivatives, and 4H-pyran-4-one derivative. Diketopiperazines were prominent in the tentative GC-MS profile and are plausible candidates for further study. However, the present data do not establish a causal contribution of this class or a correlation between diketopiperazine abundance and apoptosis. Activity-guided fractionation, authentic standards, and testing of isolated fractions or compounds are required. Oh et al. demonstrated that CFS of *Odoribacter splanchnicus* exhibited anti-colorectal cancer activity, and their GC-MS analysis similarly identified malic acid and other organic compounds as potential active molecules (Oh et al., 2021). While their study identified different compounds (malic acid, L-alanine, L-proline), the methodological approach of using GC-MS to characterize bioactive metabolites from gut microbiota CFS validates the analytical strategy employed in the current study.

Diketopiperazine reports have been common in *Lactobacillus* spp. reported 16 structurally characterized cyclic dipeptides in *Lactobacillus plantarum* which indicates that the compounds are central secondary metabolites of lactic acid bacteria (Liu et al., 2025b; Choi et al., 2011; Dezaki et al., 2024). The appearance of organic acids such as octadecanoic and hexadecanoic acid and esters in CFS profile is in agreement with known metabolic functionalities of lactobacilli. Bianchi et al. reported that *L. rhamnosus* CFS contained most of the lactic acid and was primarily responsible for antibacterial and antibiofilm activity against *Pseudomonas aeruginosa* (Bianchi et al., 2025). Their proteomic analysis also identified cell wall hydrolases, ABC transporter proteins, and enzymes that may contribute to the bioactive properties of CFS, supporting the notion that multiple components work synergistically.

The observed cytotoxic activity likely results from synergistic interactions among multiple compound classes (diketopiperazines, organic acids, heterocyclic compounds) rather than a single dominant metabolite. The aromatic and heterocyclic compounds detected, including indole derivatives, pyrazines, and 4H-pyran-4-one derivatives, may also contribute to the overall bioactivity (Dehghani et al., 2021). Sharma et al. characterized metabolites from *Lactobacillus rhamnosus* MD 14 and identified similar compound classes, including organic acids and heat-sensitive proteins, which exhibited 70%–80% cytotoxicity against HT-29 colon cancer cells and Caco-2 (Sharma et al., 2020). The selective cytotoxicity to malignant cells demonstrated by LGG-derived CFS reported (Vivarelli et al., 2019) is especially remarkable; even in the highest concentrations of LGG-SN (90 v/v), the non-transformed fibroblasts maintained 96.5% viability. However, we did not test LGG-CFS on non-transformed colon epithelial cells (e.g., CCD-841 CoN) in the current study; this represents an important limitation, as selectivity for cancer cells over normal cells is a critical criterion for potential therapeutic agents. This is consistent with reports of selective cytotoxicity of LGG- and related *Lactobacillus*-derived CFS/postbiotics toward malignant *versus* non-transformed cells in other model systems (Salemi et al., 2023; Banakar et al., 2023), supporting the biological plausibility of a selective effect, though direct confirmation in the present HT-29/CCD-841 CoN system is required.

The concentration-dependent cytotoxicity observed in this study, with an IC50 of 14.2 μg/mL against HT-29 cells, demonstrates potent anti-proliferative activity. For quantitative comparison: Salemi et al. (2023) reported that LGG cell-free supernatant selectively reduced viability of cancer cells (HCT-116, Caco-2, HT-29, and A375 melanoma) in a concentration-dependent manner while showing no effect on non-transformed fibroblasts, with IC50 values ranging from 73.8% to 91.8% (v/v) LGG-SN depending on cell line. Sharma et al. (2020) reported that CFS from four LAB isolates exhibited 70%–80% cytotoxicity against HT-29 and Caco-2 cells, increasing to 80%–90% upon extraction and formulation. Dehghani et al. (2021) reported that *L. rhamnosus* supernatant induced 58.2% and 35.35% apoptosis in HT-29 cells after 48 and 72 h, respectively. The higher apoptosis percentage observed in the current study (47.6% early apoptosis) may reflect differences in CFS preparation (MRS-based vs. RPMI-based), concentration normalization (total solids vs. % v/v), or cell culture conditions.

Review of the molecular processes of the positive effect of post biotics, highlighted the fact that anti-proliferative effects of exopolysaccharides, peptides, short-chain fatty acids and proteins are the postbiotic metabolites that activate various signalling pathways (Rad et al., 2021). The high early apoptosis rate (47.6%) observed with minimal necrosis (2.3%) in the present study indicates that cell death occurs via programmed apoptotic pathways rather than non-specific cytotoxic injury. This finding is consistent with Salemi et al. (2023), who used cleaved caspase-3 Western blot, PARP cleavage analysis, and Annexin V/PI flow cytometry to demonstrate that LGG-SN induces apoptosis in cancer cells. However, we did not measure molecular markers of apoptosis (caspase activation, Bax/Bcl-2 ratio, PARP cleavage) or assess upstream mechanisms such as mitochondrial membrane potential, oxidative stress, or DNA damage; these represent important directions for future mechanistic studies.

The molecular mechanisms underlying probiotic-induced apoptosis have been extensively characterized. An experiment demonstrated that *L. rhamnosus* supernatant caused upregulation of pro-apoptotic genes including caspase-3, Bax and caspase-9 while down-regulating anti-apoptotic Bcl-2 and genes involved in cell cycle progression (cyclin D1, cyclin E, and ERBB2). This pattern indicates activation of the intrinsic mitochondrial apoptotic pathway, where increased Bax/Bcl-2 ratio leads to mitochondrial outer membrane permeabilization, cytochrome c release, and subsequent caspase cascade activation (Dehghani et al., 2021).

Zhang et al. reported the anti-gastric cancer activity of *Lactiplantibacillus plantarum* YT013 CFS and demonstrated the BAX, BAD, and caspase-3/8/9 using flow cytometry analysis showed total apoptotic cells of 25.0%, 34.1%, and 42.6% at 200, 400, and 800 μg/mL, respectively—a dose-dependent effect consistent with the current findings (Zhang et al., 2022). The higher apoptosis percentage in the current study (47.6%) may reflect the use of colon cancer cells (which may be more responsive to gut-derived bacterial metabolites) *versus* gastric cancer cells, or differences in the specific bioactive compound profiles between LGG and *L. plantarum*.

A low rate of necrosis (2.3%) in our research is especially remarkable as it means that the cell death occurs via the programmed pathways of apoptosis rather than non-specific cytotoxic injury. The findings are consistent with the report by Salemi et al. who used several assays, such as cleaved caspase-3 Western blot, PARP cleavage analysis, and Annexin V/PI flow cytometry to prove that LGG-SN causes cytostatic G2/M arrest (Salemi et al., 2023). It might be due to the differences in CFS preparation: the authors of Salemi et al. prepared conditioned medium using RPMI-1640, and our experiments were performed in MRS-based supernatant (Salemi et al., 2023). They also observed that only MRS medium has cytotoxicity against eukaryotic cells and the choice of RPMI-1640 as their LGG conditioning media was specifically aimed at removing possible harmful elements of the MRS medium.

Abbasi et al. examined the use of post biotics based on CRL431 of the bacterium, which is a strain of *Lactobacillus casei*, and they showed using the DAPI staining that the treatment led to nuclear fragmentation and chromatin condensation of HCT-116 and HT-29 cells, which are characteristic of apoptosis (Abbasi et al., 2022).

This activity of the mitochondrial pathway is also justified by the JC -10 post-staining (Zhang et al., 2020). They demonstrated that CCS2 (the CFS of *L. plantarum*) provoked a major dose-dependent reduction of Mitochondrial Membrane Potential (MMP). The loss of MMP is an initial phenomenon that happens in intrinsic apoptosis, and it occurs before the release of cytochromec and caspase is activated. Our high diketopiperazine level (45 percent of total peak area) and high early rate of apoptosis (47.6%) are correlated, indicating that these cyclic dipeptides might be important primary mediators of activation of the mitochondrial pathway.

Cell cycle analysis of LGG-CFS treated HT-29 cells revealed a distribution of G1 (47.3%), S (38.9%), G2/M (3.22%), and sub-G1 (2.60%). Since reliable control histogram quantification was not available, direct phase-by-phase comparison was not performed. However, the relatively elevated S phase and reduced G2/M population in treated cells are suggestive of possible G1/S checkpoint involvement, consistent with reports of probiotic metabolites disrupting early cell cycle progression. As an example, S-layer protein of *Lactobacillus acidophilus* prevented the progression of HT-29 cell lines to the G1 stage by upregulating the p53, p21 and p16 (Zhang et al., 2020). Equally, CFS of *Pediococcus pentosaceus* OBK05 prevented G1 to S phase transition in cell line, and its IC50 was 54.51 μg/mL (Bhukya and Bhukya, 2023). These results, together with the G0/G1 arrest caused by *Lactobacillus delbrueckii* supernatant of HT-29 cells, indicate that anti-proliferative actions of probiotic metabolites are usually mediated by the disruption of early cell cycle progression (Pahlavianiet al., 2025).

Abbasi et al. reported that *L. casei* CRL431 post biotics arrested HCT-116 cell cycle in sub-G1 (G0/G1) phase (80.56% ± 6.2%) and G2/M phase (12.6% ± 3.7%), indicating that different *Lactobacillus* species and strains may target different cell cycle checkpoints (Abbasi et al., 2022). The predominance of sub-G1 accumulation in their study (indicating apoptosis) combined with G2/M arrest suggests multiple mechanisms of action.

The cell-cycle analysis of LGG-CFS-treated HT-29 cells showed a distribution of 47.3% in G1, 38.9% in S, and 3.22% in G2/M, suggesting an altered cell-cycle distribution following treatment. However, because reliable quantitative control data were unavailable, the possibility of specific G1/S checkpoint disruption remains inferential and requires validation using properly quantified control samples and checkpoint-specific molecular markers. We acknowledge that without checkpoint-specific markers (e.g., cyclin D1/E expression, p21/p27, phospho-Rb), the interpretation of G1/S disruption remains inferential. This pattern differs from some reports but is consistent with others, highlighting strain-specific and preparation-specific effects. Our finding of G1/S accumulation differs from Salemi et al. (2023), who reported cytostatic G2/M arrest with LGG-SN; this discrepancy may reflect differences in CFS preparation methodology (MRS-based vs. RPMI-based supernatants) and highlights the need for standardized preparation protocols.

The concentrations used throughout this study were normalized to the total mass of freeze-dried LGG-CFS obtained from sterile neutralized culture supernatant. This approach was selected because the biological activity of probiotic-derived post biotics is generally attributed to the combined action of multiple metabolites rather than a single purified compound. Consequently, the complete lyophilized preparation was evaluated as the experimental test material. Nevertheless, we acknowledge that total lyophilized solids also include residual constituents derived from the culture medium, making direct quantitative comparison with studies reporting protein-normalized preparations or purified metabolite fractions difficult. Future studies should therefore quantify total protein content and major bioactive metabolites, followed by activity-guided fractionation, to establish standardized potency metrics and improve inter-study comparability.

Several important limitations of this study must be acknowledged. First, the MTT assay alone is insufficient to fully characterize cell death; orthogonal assays (e.g., trypan blue exclusion, ATP-based viability, clonogenic survival) would strengthen viability assessments. Second, the absence of a non-transformed colonic epithelial cell line (e.g., CCD-841 CoN) limits conclusions about selective cytotoxicity. Future studies will evaluate LGG-CFS using normal human colon epithelial cell lines (CCD-841 CoN or NCM460) to determine therapeutic selectivity and establish a safety index. Third, the lack of molecular markers for apoptosis (caspases, Bcl-2 family) and cell cycle checkpoints (cyclins, CDKs, CDK inhibitors) limits mechanistic insight. Fourth, the concentrations used in this study were normalized to total lyophilized LGG-CFS solids rather than to total protein content or specific metabolite concentrations. Although this strategy provides reproducible dosing of the complete post biotic preparation, the presence of residual culture-medium components limits direct comparison of the reported IC_50_ values with studies using purified protein fractions or defined metabolite concentrations. Future work should include protein quantification, metabolomic normalization, and activity-guided fractionation to identify the principal bioactive constituents. Fifth, the GC-MS identifications are tentative without authentic standards, and causal attribution of bioactivity to specific compounds requires activity-guided fractionation. Sixth, a single time point (24 h) was used; time-course experiments would help distinguish acute toxicity from progressive growth inhibition, consistent with the time-dependent response pattern previously reported for *L. rhamnosus* supernatant on HT-29 cells (Dehghani et al., 2021). Seventh, only one colorectal cancer cell line (HT-29) was tested; additional lines with different genetic backgrounds (e.g., HCT-116 with KRAS mutation, Caco-2) would improve generalizability. Finally, we did not include a separate acidified medium control to rule out pH effects, although the CFS was neutralized to pH 7.2–7.4 prior to treatment.

## 5 Conclusion

LGG-CFS demonstrated dose-dependent cytotoxic effects against HT-29 colorectal cancer cells with an IC_50_ of 14.2 μg/mL. Mechanistic studies revealed that the anti-proliferative effect is mediated through induction of early apoptosis (Annexin V^+^/PI^−^), with flow cytometry confirming 47.6% of cells transitioning to early apoptotic state after treatment and minimal necrotic cell death (2.3%). Cell-cycle analysis of LGG-CFS-treated HT-29 cells showed a distribution of 47.3% in G1 and 38.9% in S phase, with 3.22% in G2/M and 2.60% in the sub-G1 fraction. This distribution suggests an alteration in cell-cycle progression; however, specific G1/S checkpoint involvement could not be statistically confirmed because reliable quantitative control data were unavailable. GC-MS profiling revealed a complex metabolite profile with diketopiperazines as the predominant class, which may contribute to the observed bioactivity, though causal validation requires activity-guided fractionation. The present findings are preliminary and limited to *in vitro* observations in a single colorectal cancer cell line. The correlation between metabolite profiles and biological activity is supported by existing literature indicating that *L. rhamnosus* GG-derived metabolites have anticancer effects through various mechanisms, including induction of apoptosis and regulation of cell cycle progression. However, the following critical gaps must be addressed before translational conclusions can be drawn: (1) testing on non-transformed colonic epithelial cells to assess selectivity; (2) validation in additional colorectal cancer lines with different genetic backgrounds; (3) mechanistic studies including molecular markers of apoptosis (caspase activation, Bax/Bcl-2 ratio) and cell cycle regulation (cyclins, CDKs); (4) activity-guided fractionation to identify specific bioactive compounds; (5) *in vivo* efficacy and safety studies; and (6) assessment of potential interactions with standard chemotherapeutic agents. Given these limitations, we conclude that LGG-CFS warrants further investigation as a candidate adjunctive agent for colorectal cancer, but current evidence is restricted to *in vitro* findings and does not support clinical or translational claims. The preferential induction of apoptosis with minimal necrosis and the presence of multiple bioactive compound classes that may act synergistically provide a rationale for continued investigation. Because the reported concentrations represent total lyophilized CFS rather than purified bioactive fractions, the IC_50_ values should be interpreted as preparation-specific and not as absolute concentrations of individual active metabolites. Future research must focus on isolation and characterization of pure diketopiperazine compounds, elucidation of their mechanisms of action, confirmation of efficacy in animal models, and assessment of selectivity using normal colon epithelial cells.

## Data Availability

The raw data supporting the conclusions of this article will be made available by the authors, without undue reservation.

## References

[B1] Abbasi A. Homayouni Rad A. Ghasempour Z. Sabahi S. Samadi Kafil H. Hasannezhad P. (2022). The biological activities of postbiotics in gastrointestinal disorders. Crit. Rev. Food Sci. Nutr. 62 (22), 5983–6004. 10.1080/10408398.2021.1895061 33715539

[B2] Atarashi K. Tanoue T. Shima T. Imaoka A. Kuwahara T. Momose Y. (2011). Induction of colonic regulatory T cells by indigenous *Clostridium* species. Science 331 (6015), 337–341. 10.1126/science.1198469 21205640 PMC3969237

[B3] Banakar M. Etemad-Moghadam S. Haghgoo R. Mehran M. Yazdi M. H. Mohamadpour H. (2023). Anticancer activity of postbiotic mediators derived from *Lactobacillus rhamnosus* GG and *Lactobacillus reuteri* on acute lymphoblastic leukemia cells. Galen. Med. J. 12, e3096. 10.31661/gmj.v12i.3096 38774842 PMC11108661

[B4] Banna G. L. Torino F. Marletta F. Santagati M. Salemi R. Cannarozzo E. (2017). *Lactobacillusrhamnosus*GG: an overview to explore the rationale of its use in cancer. Front. Pharmacol. 8, 603. 10.3389/fphar.2017.00603 28919861 PMC5585742

[B5] Bhukya K. K. Bhukya B. (2023). Exploration of antidiabetic, cholesterol-lowering, and anticancer upshot of probiotic bacterium *Pediococcuspentosaceus* OBK05 strain of buttermilk. Probiotics Antimicrob. Proteins 15 (6), 1484–1500. 10.1007/s12602-022-10002-0 36282441

[B6] Bianchi M. Kaya E. Logiudice V. Maisetta G. Curtis A. Kavanagh K. (2025). Biotherapeutic potential of different fractions of cell-free supernatants from *Lactobacillusrhamnosus*against *Pseudomonas aeruginosa* . Front. Cell. Infect. Microbiol. 15, 1608897. 10.3389/fcimb.2025.1608897 40661974 PMC12256502

[B7] Capurso L. (2019). Thirty years of *Lactobacillusrhamnosus*GG: a review. J. Clin. Gastroenterol. 53, S1–S41. 10.1097/mcg.0000000000001170 30741841

[B8] Choi E. J. Park J. S. Kim Y. J. Jung J. H. Lee J. K. Kwon H. C. (2011). Apoptosis-inducing effect of diketopiperazine disulfides produced by *Aspergillus* sp. KMD 901 isolated from marine sediment on HCT116 colon cancer cell lines. J. Applied Microbiology 110 (1), 304–313. 10.1111/j.1365-2672.2010.04885.x 21122037

[B9] Dehghani N. Tafvizi F. Jafari P. (2021). Cell cycle arrest and anti-cancer potential of probiotic *Lactobacillusrhamnosus*against HT-29 cancer cells. BioImpacts 11 (4), 245–252. 10.34172/bi.2021.32 34631486 PMC8494254

[B10] Dezaki F. S. Narimani T. Ghanadian M. Bidram E. Poursina F. (2024). Antimicrobial and antibiofilm effects of cyclic dipeptide-rich fraction from *Lactobacillus plantarum* loaded on graphene oxide nanosheets. Front. Microbiol. 15, 1391039. 10.3389/fmicb.2024.1391039 39286346 PMC11402667

[B11] Drago L. (2019). Probiotics and colon cancer. Microorganisms 7 (3), 66. 10.3390/microorganisms7030066 30823471 PMC6463067

[B12] Gagnière J. Raisch J. Veziant J. Barnich N. Bonnet R. Buc E. (2016). Gut microbiota imbalance and colorectal cancer. World J. Gastroenterol. 22 (2), 501–518. 10.3748/wjg.v22.i2.501 26811603 PMC4716055

[B13] Ghasemi M. Turnbull T. Sebastian S. Kempson I. (2021). The MTT assay: utility, limitations, pitfalls, and interpretation in bulk and single-cell analysis. Int. J. Mol. Sci. 22 (23), 12827. 10.3390/ijms222312827 34884632 PMC8657538

[B14] Hossain M. S. Karuniawati H. Jairoun A. A. Urbi Z. Ooi D. J. John A. (2022). Colorectal cancer: a review of carcinogenesis, global epidemiology, current challenges, risk factors, preventive and treatment strategies. Cancers 14 (7), 1732. 10.3390/cancers14071732 35406504 PMC8996939

[B15] Liu R. Sun B. Kang S. O. Hong Y. Yeo J. Y. Wang J. H. (2025a). The combined application of cis-cyclo (L-Phe-L-Pro) with antimicrobial proline-based 2, 5-diketopiperazines significantly enhances anti-breast cancer activity by selectively targeting cancer stem cells. Chemico-Biological Interact. 417, 111565. 10.1016/j.cbi.2025.111565 40409392

[B16] Liu R. Shin G. No Y. Shin J. Kang S. Park P. (2025b). Optimization of the culture conditions of lactic acid bacteria for antimicrobial activity and mass production of Cyclic dipeptides. J. Microbiology Biotechnology 35, e2408007. 10.4014/jmb.2408.08007 40537898 PMC12197806

[B17] Martínez-Maqueda D. Miralles B. Recio I. (2015). “HT29 cell line,” in The Impact of Food Bioactives on Health: In Vitro and Ex Vivo Models. Editor Verhoeckx K. (Springer), 113–124. 10.1007/978-3-319-16104-4_11 29787039

[B18] Morita H. Toh H. Oshima K. Murakami M. Taylor T. D. Igimi S. (2009). Complete genome sequence of the probiotic *Lactobacillusrhamnosus*ATCC 53103. J. Bacteriol. 191 (24), 7630–7631. 10.1128/JB.01287-09 19820099 PMC2786603

[B19] Oh B. S. Choi W. J. Kim J. S. Ryu S. W. Yu S. Y. Lee J. S. (2021). Cell-free supernatant of *Odoribactersplanchnicus* isolated from human feces exhibits anti-colorectal cancer activity. Front. Microbiol. 12, 736343. 10.3389/fmicb.2021.736343 34867852 PMC8638082

[B20] Pahlaviani M. R. M. K. Emami A. Mahmoodi S. M. M. Rezaeian A. A. (2025). Evaluation of Lactobacillus delbrueckii supernatant effects on cell cycle regulation, apoptosis induction, and gene expression in HT-29 colorectal cancer cells. Int. J. Mol. Cell. Med. 14 (3), 872–885. 10.22088/IJMCM.BUMS.14.3.872 41321967 PMC12664687

[B21] Pozarowski P. Darzynkiewicz Z. (2004). “Analysis of cell cycle by flow cytometry”. in Checkpoints Controls Cancer Methods in Molecular Biology Schönthal A. H. , Totowa, NJ: Humana Press. 281. 301–311. 10.1385/1-59259-811-0:301 15220539

[B22] Rad A. H. Aghebati-Maleki L. Kafil H. S. Abbasi A. (2021). Molecular mechanisms of postbiotics in colorectal cancer prevention and treatment. Crit. Rev. Food Sci. Nutr. 61 (11), 1787–1803. 10.1080/10408398.2020.1765310 32410512

[B23] Salemi R. Vivarelli S. Ricci D. Scillato M. Santagati M. Gattuso G. (2023). *Lactobacillus rhamnosus*GG cell-free supernatant as a novel anti-cancer adjuvant. J. Transl. Med. 21 (1), 195. 10.1186/s12967-023-04036-3 36918929 PMC10015962

[B24] Segers M. E. Lebeer S. (2014). Towards a better understanding of *Lactobacillusrhamnosus*GG-host interactions. Microb. Cell Factories 13 (1), S7. 10.1186/1475-2859-13-S1-S7 25186587 PMC4155824

[B25] Sharma M. Chandel D. Shukla G. (2020). Antigenotoxicity and cytotoxic potentials of metabiotics extracted from isolated probiotic, *Lactobacillusrhamnosus*MD 14 on Caco-2 and HT-29 human colon cancer cells. Nutr. Cancer 72 (1), 110–119. 10.1080/01635581.2019.1615514 31266374

[B26] Taieb J. Gallois C. (2020). Adjuvant chemotherapy for stage III colon cancer. Cancers 12 (9), 2679. 10.3390/cancers12092679 32961795 PMC7564362

[B27] Thursby E. Juge N. (2017). Introduction to the human gut microbiota. Biochem. Journal 474 (11), 1823–1836. 10.1042/BCJ20160510 28512250 PMC5433529

[B28] Vivarelli S. Salemi R. Candido S. Falzone L. Santagati M. Stefani S. (2019). Gut Microbiota and cancer: from pathogenesis to therapy. Cancers 11 (1), 38. 10.3390/cancers11010038 30609850 PMC6356461

[B29] Xie Y. H. Chen Y. X. Fang J. Y. (2020). Comprehensive review of targeted therapy for colorectal cancer. Sig Transduct. Target Ther. 5, 22. 10.1038/s41392-020-0116-z 32296018 PMC7082344

[B30] Zhang T. Pan D. Yang Y. Jiang X. Zhang J. Zeng X. (2020). Effect of *Lactobacillus acidophilus* CICC 6074 S-layer protein on colon cancer HT-29 cell proliferation and apoptosis. J. Agricultural Food Chemistry 68 (9), 2639–2647. 10.1021/acs.jafc.9b06909 32000489

[B31] Zhang R. Zhou Z. Ma Y. Du K. Sun M. Zhang H. (2022). Anti-gastric cancer activity of the cell-free culture supernatant of serofluid dish and *Lactiplantibacillus plantarum* YT013. Front. Bioeng. Biotechnol. 10, 898240. 10.3389/fbioe.2022.898240 35677304 PMC9168531

