# Correction: *In vitro* cytotoxic and pro-apoptotic effects of *Lactobacillus* rhamnosus GG cell-free supernatant on HT-29 colorectal cancer cells

**DOI:** 10.3389/fmolb.2026.2005518

**Published:** 2026-10-12

**Authors:** Frontiers Production Office

**Affiliations:** Frontiers Media SA, Lausanne, Switzerland

**Keywords:** apoptosis assay, cell cycle, GC-MS analysis, HT-29 cell line, *Lactobacillus* rhamnosus GG, MTT assay

In the published article there was an error in the affiliation of reviewer Ali Miri. This has now been corrected to Baqiyatallah Medical Sciences University, Iran.

The original version of this article has been updated.

